# Naturally Acquired *Plasmodium knowlesi* Malaria in Human, Thailand

**DOI:** 10.3201/eid1012.040293

**Published:** 2004-12

**Authors:** Somchai Jongwutiwes, Chaturong Putaporntip, Takuya Iwasaki, Tetsutaro Sata, Hiroji Kanbara

**Affiliations:** * Chulalongkorn University, Bangkok, Thailand;; † Institute of Tropical Medicine, Nagasaki University, Nagasaki,; ‡ National Institute of Infectious Diseases, Tokyo, Japan

**Keywords:** Plasmodium knowlesi, simian malaria, primate, small subunit ribosomal RNA, mitochondrial cytochrome b, naturally acquired infection, sequence, Giemsa stain, Thailand, dispatch

## Abstract

We describe a case of naturally acquired infection with *Plasmodium knowlesi* in Thailand. Diagnosis was confirmed by the small subunit ribosomal RNA and the mitochondrial cytochrome b sequences. The occurrence of simian malaria in human has signified the roles of wild primate populations in disease transmission in some malaria-endemic areas.

A number of emerging pathogens have been known to cross-transmit between humans and nonhuman hosts. Wild primate populations have the potential to serve as origins and reservoirs of certain human pathogens, ranging from virus to helminths (*1*). More than 26 species of *Plasmodium* circulate among primate populations (*2*). Several of the simian malaria species are closely related to the human ones, and some of these, e.g. *Plasmodium simium*, *P. brasilianum*, *P. cynomolgi*, *P. inui*, and *P. knowlesi*, have been implicated in symptomatic malaria in humans in experimental, accidental, or natural infections (*2**–**7*). Before the advent of molecular tools for diagnosing infectious diseases, identifying simian malaria in humans required expertise in the structure of these parasites, experimental studies in mosquito vectors, and tests for infectivity to primate hosts (*6**,**7*). In general, simian malaria is not included in the differential diagnosis of human infections, which could partly stem from lack of awareness about the zoonotic potential of these parasites. Furthermore, the current laboratory methods for species differentiation target only the four human plasmodia species. On the other hand, simian malaria species that display structural similarity to those species commonly found in humans may be unnoticed in routine examinations of blood smears. We describe a patient who acquired *P. knowlesi* infection while staying in a forest in southern Thailand where human malaria is endemic.

## The Study

In August 2000, a 38-year-old Thai man came to an outpatient department of King Chulalongkorn Memorial Hospital, Bangkok, with daily fever, headache, intermittent chill, sweating, and malaise for 4 days. His home was in a suburb of Bangkok, where no malaria transmission has been reported. During the past few months before the present illness, he spent several few weeks in a hilly forest area in Prachuap Khiri Khan Province in southern Thailand, ≈300 km from Bangkok near the Thai-Myanmar border. He reported having fever 1 week after returning home. He did not know of any underlying illness and had not experienced any previous malaria attacks. Although he stayed in a cottage and slept inside a mosquito net, he remembered being bitten frequently by mosquitoes, especially at dusk and dawn.

Upon examination, his temperature was 38.5°C, and pulse rate was 90 beats per minute. His hemoglobin was 14.0 g/dL, hematocrit was 0.4, and erythrocyte count was 4.2 x 10^6^ cells/μL. The total leukocyte count was 5,500 cells/μL, with normal differential count. The platelet count was 90,000/μL. Levels of other laboratory investigations, including urinalysis, blood sugar, liver function test, blood urea nitrogen, and creatinine, were normal. Examination of Giemsa-stained thin blood films showed 10% young trophozoites, 45% growing trophozoites, 40% schizonts, and 5% gametocytes (n = 300). The parasite structure was compatible with that of *P. malariae*. The parasite density inferred from the number of malarial parasites per 500 leukocytes in thick blood smear yielded 1,155/μL or equivalent to parasitemia 0.03%. The patient was treated with 10 mg/kg of oral chloroquine initially, followed by 5 mg/kg, 6 hours later on the day 1, and 5 mg/kg/day for the next 2 days. On day 2, with a temperature of 37.5°C, he came to the hospital. Parasitemia decreased to 137/μL. Complete defervescence was observed on day 3, and parasitemia could not be detected. Two weeks and 2 months later, his blood smears were negative for malaria. Fever did not recur.

**Figure 1:**
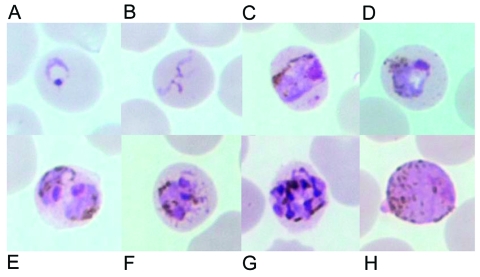
Giemsa-stained thin blood films depicting A) ring stage, B) tenue form of young trophozoite, C) band-shaped growing trophozoite, D) growing trophozoite with little or no amoeboid activity, E) double growing trophozoites, F) early schizont, G) late schizont in an erythrocyte with fimbriated margins, and H) mature macrogametocyte. Discernible Sinton and Mulligan stippling is in C, D, and F.

Meanwhile, we recently evaluated a DNA-based diagnostic method by the polymerase chain reaction (PCR) targeting the small subunit ribosomal RNA (SSU rRNA) genes of all four species of human malaria as reported (*8*). Ten isolates each for *P. falciparum*, *P. vivax*, and *P. malariae* and four isolates of *P. ovale* were used as positive controls. Results showed that all isolates gave concordant positive PCR products with those diagnosed by microscopy except an isolate from this patient (data not shown). Retrospective examination of blood smears has shown several developmental stages of malaria parasites similar to those typically seen in *P. malariae*. However, some erythrocytes that harbored mature asexual parasites possessed fimbriated margins. The cytoplasm of some young trophozoites appeared spread out into the network of irregular pseudopodia, and the chromatin was distributed into fragments, conforming to the tenue forms. Pinkish dots varying from fine to large irregular masses called Sinton and Mulligan's stippling developed intracorpuscularly with the maturation of some parasites (Figure 1).

To elucidate the species of malaria infecting our patient, we determined the SSU rRNA gene by using similar methods as described by others (*9*), except that ExTaq DNA polymerase (Takara, Japan), pGEM-T vector (Promega, USA), and *Escherichia coli* strain JM109 were used. Results showed that the SSU rRNA sequence contained 97.8% to 99.6% homology with those of *P. knowlesi* transcribed during asexual stages or the type A gene (GenBank accession no. AY327549-AY327557, L07560, and U72542) (*3**,**9*). Nucleotide sequence data reported in this study are available in the EMBL, GenBank, and DDJB databases under the accession no. AY580317–8.

**Figure 2:**
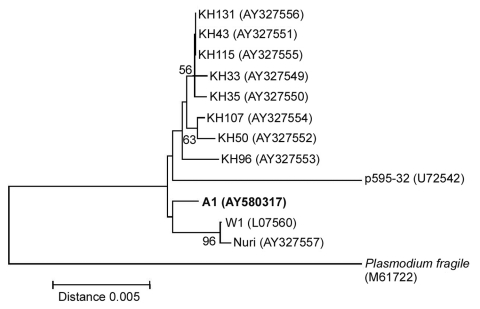
Neighbor-joining tree based on the asexually transcribed SSU rRNA sequences displaying the phylogenetic position of isolate A1 in this study in relation to other *Plasmodium knowlesi* isolates (AY327549-AY327556 from humans, and L07560, U72542, and AY327557 from monkeys) and *P. fragile* (M61722). The tree was constructed with Kimura's two-parameter distance. including transitions and transversions as implemented in the MEGA version 2.1 software. Bootstrap percentages more than 50% based on 1,000 replicates are shown on the branches.

Phylogenetic tree showed that *P. knowlesi* in this study was closely related to the W1 and Nuri strains, although its divergence from Malaysian human isolates was not supported by bootstrap analysis (Figure 2). Consistently, the mitochondrial cytochrome b gene of this isolate, determined by the methods similar to previous report except the PCR primers (mtPk-F:5´-AGGTATTATATTCTTTATACAAATATTAAC-3´ and mtPk-R:5´-TCTTTTATAATGAACAAGTGTAAATAATC-3´), displayed perfect sequence identity with that of *P. knowlesi* strain H from monkey (AF069621) (*4*).

## Conclusions

*P. knowlesi* is prevalent among crab-eating macaques, *Macaca fascicularis*, in the Malaysian peninsula and the Philippines (*2**,**10*). Other known natural hosts include pig-tailed macaques, *M. nemestrina*, and leaf monkeys, *Presbytis melalophos* (*2**,**10*). Although in 1932, Knowles et al. (*11*) had shown that *P. knowlesi* isolated from monkey could be infectious to humans, the first naturally acquired human infection with *P. knowlesi* was not reported until 1965 (*6*); the patient was infected in a Malaysian forest. In 1971 the second case, albeit presumptive, occurred in a man who also acquired the infection in a forest in Malaysia (*12*). Recently, a large cluster of human infections caused by *P. knowlesi* has been identified from Malaysian Borneo (*9*). Our report has expanded the geographic range for natural transmission of *P. knowlesi* to a forest in Thailand near southern Myanmar border, where wild populations of crab-eating macaques, despite being considered endangered, are still substantial.

The prevalence of naturally acquired primate malaria in humans can be underestimated from examination of blood films. The reported abundance of ring stages of *P. knowlesi* found in the first naturally acquired human case led to the initial diagnosis of *P. falciparum*, while the mature parasites could masquerade as those of *P. malariae*, as we encountered in this patient (*6*). Although structural descriptions of young trophozoites of *P. knowlesi* have been delineated, we were unable to find the ring form with double chromatin dots (*9*). Conversely, a few young trophozoites resembled the tenue forms, proposed by Stephens in 1914 (*13*) to be a distinct species. However, the tenue form has recently been recognized to be a *P. malariae* variant found in Myanmar (*8*). The presence of the tenue form in the blood smears of our patient, despite the low number, rather suggests a shared structural feature among species of malaria. The possibility of coinfection between *P. knowlesi* with one or more of the four human malaria species was not supported by our PCR detection. The structure of *P. knowlesi* is highly dependent on the host erythrocytes, i.e., resembling *P. vivax* in *M. fascicularis*, *P. falciparum* in rhesus monkeys, and *P. malariae* in humans (*2**,**9**,**11**,**12*). Although stippling was not seen among *P. knowlesi*–infected blood smears of Sarawak's patients, the presence of Sinton-Mulligan stippling in infected erythrocytes in this study is in accord with the report by Fong et al., in which erythrocytic stippling served as one of the diagnostic feature (*9**,**12*). Such discrepancy could partly arise from differences in the condition for Giemsa staining, infecting parasite strains, or both.

The complete asexual erythrocytic cycle of *P. knowlesi* in human and its natural macaque host requires ≈24 hours, coinciding with a quotidian fever pattern. However, fever pattern per se may not be a precise indicator for differentiating malaria caused by *P. knowlesi* and *P. malariae*. Although the merogony cycle of *P. malariae* has been generally known to be 72 hours, fever patterns might not be strictly quartan (*14*). Meanwhile, the preexisting immunity to *P. vivax* has reportedly conferred partial resistance to induced infection during malariotherapy (*2*). Whether naturally acquired immunity against *P. vivax* can reduce symptoms in *P. knowlesi* infection requires further investigation.

To date, little is known about the extent of variation in the *P. knowlesi* population. Analysis of the SSU rRNA gene from the isolate in this study has shown minor difference from those of *P. knowlesi* from monkeys and patients in Malaysian Borneo (*3**,**9*). Evidence from malariotherapy showed that *P. knowlesi* could lose or increase its virulence on blood passage in humans, which suggests that strain difference could occur in wild populations and might effect humans differently (*2*). In conclusion, *P. knowlesi* could contribute to the reemergence of simian malaria in Thailand and southeast Asia, where its vectors, *Anopheles leucosphyrus* group, are abundant (*15*).
